# Sustained Cytotoxicity of Wogonin on Breast Cancer Cells by Encapsulation in Solid Lipid Nanoparticles

**DOI:** 10.3390/nano8030159

**Published:** 2018-03-13

**Authors:** Jong-Suep Baek, Young-Guk Na, Cheong-Weon Cho

**Affiliations:** College of Pharmacy and Institute of Drug Research and Development, Chungnam National University, 99 Daehak-ro, Yuseong-gu, Daejeon 34134, Korea; baekjs@cnu.ac.kr (J.-S.B.); youngguk@cnu.ac.kr (Y.-G.N.)

**Keywords:** wogonin, solid lipid nanoparticles, MCF-7, sustained release, breast cancer

## Abstract

While wogonin has been known to have cytotoxicity against various cancer cells, its bioavailability and cytotoxicity are low due to its low water solubility. Therefore, wogonin-loaded solid lipid nanoparticles were fabricated using a hot-melted evaporation technique. The highest solubility of wogonin was observed in stearic acid. Hence, wogonin-loaded solid lipid nanoparticles were composed of stearic acid as the lipid matrix. The physicochemical properties of the wogonin-loaded solid lipid nanoparticles were evaluated by dynamic laser scattering and scanning electron microscopy. The wogonin-loaded solid lipid nanoparticles exhibited sustained and controlled release up to 72 h. In addition, it was observed that the wogonin-loaded solid lipid nanoparticles exhibited enhanced cytotoxicity and inhibited poly (ADP-ribose) polymerase in MCF-7 breast cancer cells. Overall, the results indicate that wogonin-loaded solid lipid nanoparticles could be an efficient delivery system for the treatment of breast cancer.

## 1. Introduction

Wogonin (5,7-dihydroxy-8-methoxy flavone) is one of the active compounds of the flavonoids isolated from the radix of *Scutellaria baicalensis* and possesses anticancer properties in a wide range of human cancer cell lines by inducing apoptosis in vitro [1,2,3,4]. A few studies have reported that wogonin (10–30 µmol/L) allows for enhanced cellular uptake of anticancer drugs in a few cancer cell lines [5,6]. In addition, wogonin is reported to minimize apoptosis caused by anticancer drugs in normal cells, such as thymocytes [7]. Although wogonin has several advantages for treating cancer, it has major limitations such as solubility in water and, therefore, low bioavailability. Due to this limitation, wogonin is restricted in clinical administration. Recently, a few studies directed to resolving this problem have used nanoparticles, such as magnetic nanoparticles [8,9,10].

Solid lipid nanoparticles (SLNs) are nano-sized particle carriers that consist of a biocompatible and biodegradable solid lipid matrix that remains in the solid state in the human body [11,12,13,14]. SLNs have been widely developed and used for oral, ocular, intravenous, pulmonary, and intranasal administration [15,16,17,18] because of their unique benefits, such as high encapsulation efficiency, sustained and controlled release, and biodegradability [19,20,21,22]. 

The aim of this study was to develop and optimize the wogonin-loaded solid lipid nanoparticle (W-SLN). W-SLNs were able to encapsulate and release wogonin efficiently to MCF-7 breast cancer cells. It was highlighted that wogonin from W-SLNs can be released in a controlled manner to achieve a sustained cytotoxic effect on MCF-7 cells. 

## 2. Results and Discussion

### 2.1. Solubility of Wogonin in Different Lipids

**Figure 1:**
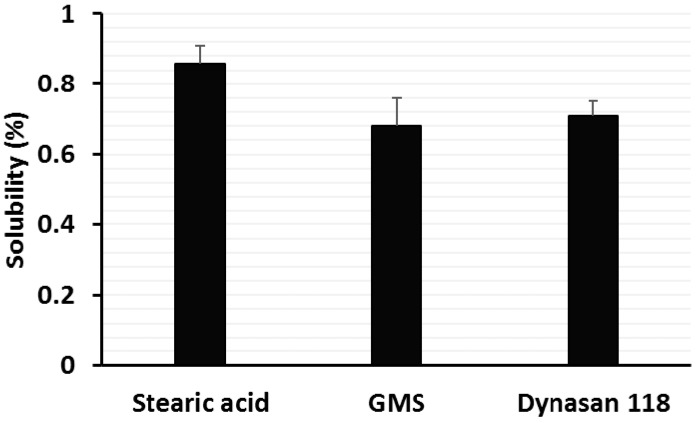
Solubility of wogonin in different solid lipids (*n* = 3, mean ± SD). GMS, glycerol monostearate.

The solubility of wogonin in the lipid matrix is a key point for determining drug loading and encapsulation efficiency in SLN. For this purpose, the solubility of wogonin in three different solid lipids was determined to maximize the drug loading. As seen in Figure 1, the solubility of wogonin in stearic acid, glyceryl monostearate (GMS), and Dynasan 118 was 0.85%, 0.68%, and 0.71% (*w*/*w*), respectively. Based on these results, stearic acid was chosen as the solid lipid.

### 2.2. Preparation and Characterization of Wogonin-Loaded Solid Lipid Nanoparticles

**Figure 2:**
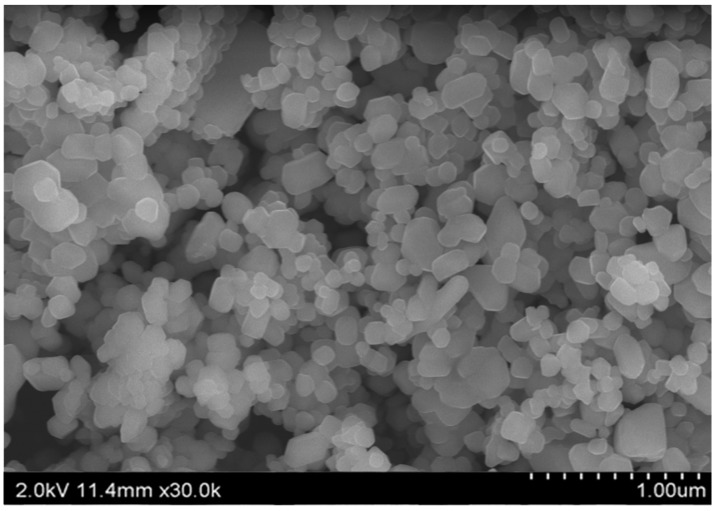
Scanning electronic microscopy (SEM) image of F2.

W-SLNs were prepared by a hot sonication method. Next, physicochemical properties such as particle size, polydispersity index, and encapsulation efficiency (E.E) were determined. The formulation variations in terms of loading amount of wogonin and their effect on the properties of W-SLNs are listed in Table 1 and Table 2. To obtain an optimal formulation in terms of particle size, polydispersity index, and E.E, the effect of the amount of wogonin on the physicochemical properties was evaluated. The particle size of the SLNs showed increasing tendency with increasing amounts of wogonin. The formula, F1, F2, and F3 showed 211.6 ± 9.2, 225.0 ± 8.3 or 243.5 ± 8.7 nm, respectively. The SEM image shows that W-SLN has a spherical morphology (Figure 2). The polydispersity index of F1 and F2 were similar, although that of F3 was higher than that of F1 and F2. The E.E (%) decreased with increasing amounts of wogonin. The E.E (%) of F3 was significantly different to the other formulations. No significant difference in zeta potential was observed in the three formulations. After considering the physicochemical properties, F2 was chosen for further studies. F2 did not show significant changes in particle size, zeta potential, and E.E (%) of wogonin after 60 days of storage at room temperature (Figure S1).

### 2.3. In Vitro Release Profiles

**Figure 3:**
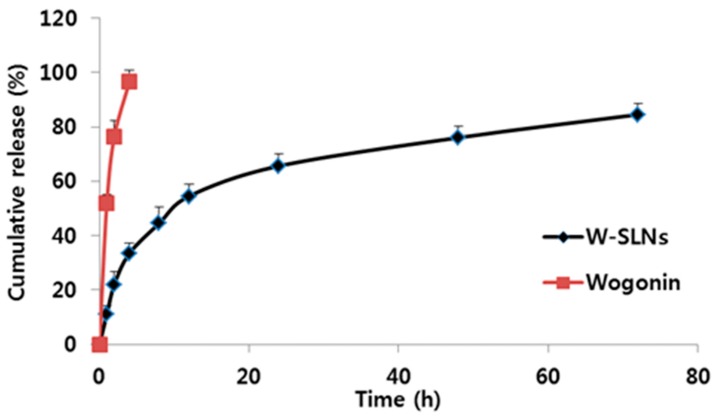
In vitro release of wogonin from wogonin-loaded solid lipid nanoparticles (W-SLNs) and wogonin solution in phosphate buffered saline (PBS) (pH 7.4, 2.0 wt % BSA) to mimic the biological environment conditions. Values represent mean ± SD (*n* = 3).

A drug release study of the wogonin solution and W-SLN was conducted (Figure 3). Wogonin solution showed complete release at only 4 h, while W-SLN exhibited a first release burst within the initial 12 h with 54.6 ± 4.5%, which was followed by a sustained release. The cumulative wogonin released from W-SLN over 72 h was 84.6 ± 4.0%.

### 2.4. Cell Growth Inhibition Study

**Figure 4:**
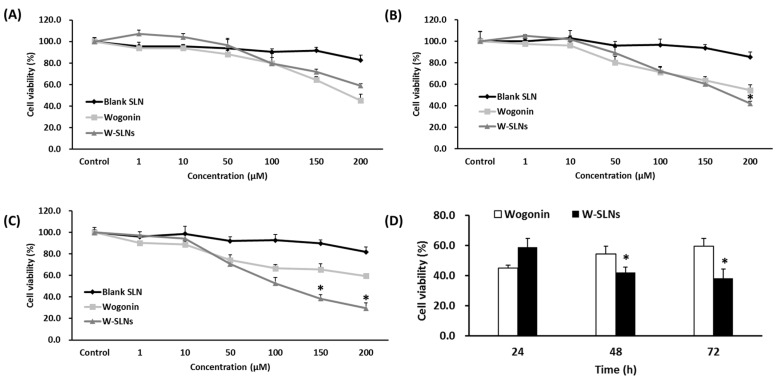
Cell viability of MCF-7 cells treated with different concentrations of blank SLN, wogonin, and W-SLN for (**A**) 24 h; (**B**) 48 h; and (**C**) 72 h. Cell viability of MCF-7 cells treated with 200 µM wogonin or W-SLN for 24, 48, and 72 h (**D**). * *p* > 0.05 shows a significant difference from wogonin.

As shown in Figure 4, there was no significant cytotoxicity of blank SLN in MCF-7 cells. Although wogonin solution exhibited significant cytotoxicity against MCF-7 cells at 24 h, the cytotoxicity did not last long. On the other hand, W-SLN showed sustained cytotoxicity against MCF-7 cells up to 72 h (*p* < 0.05). In other words, W-SLN exhibited time-dependent inhibition efficiency up to 72 h while the cell viability of MCF-7 treated with the wogonin solution was recovered over time. 

### 2.5. Cell Cycle Arrest Determined by Flow Cytometry Analysis

**Figure 5:**
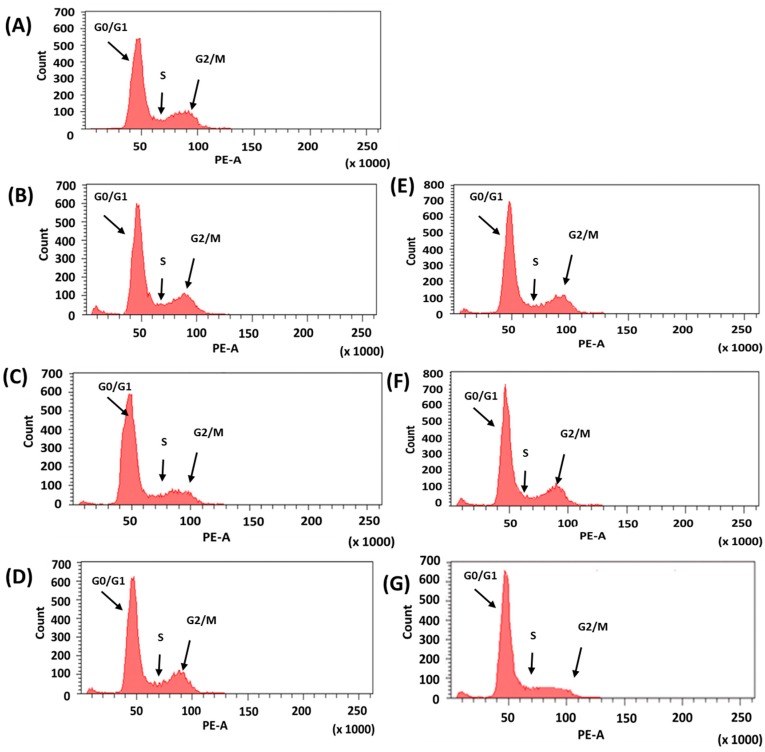
Cell cycle distribution analysis. (**A**) Control group; (**B**) wogonin for 24 h; (**C**) wogonin for 48 h; (**D**) wogonin for 72 h; (**E**) W-SLN for 24 h; (**F**) W-SLN for 48 h; and (**G**) W-SLN for 72 h (the concentration of wogonin was 200 µM). At the end of each time, cells were ethanol-fixed, stained with propidium iodide (PI), and analyzed in the DNA content by flow cytometry. Relative distribution of cell population in the cell cycle phase is presented.

In order to investigate the mechanism of wogonin-induced inhibition on cell proliferation, each cell cycle was monitored using fluorescence-activated cell sorting (FACS) to measure the cell distribution. The cell cycle after exposure to the wogonin solution or W-SLN was measured by flow cytometry at 24, 48, and 72 h. As shown in Figure 5 and Table 3, the number of cells in G0/G1 increased while the number of cells in the S phase decreased with wogonin treatment. A similar change in cell cycle was noticed in cells treated with W-SLN. Notably, the shift in cell distribution into the G0/G1 phase increased in a time-dependent manner. In addition, the ratio of the G0/G1 phase cells with W-SLN was significantly higher than those with free wogonin at 72 h. While W-SLN exhibited a decreasing tendency in the ratio of G0/G1 phase over time, the ratio of the G0/G1 phase in the cells with free wogonin recovered after 48 h.

### 2.6. Cellular Uptake Studies 

**Figure 6:**
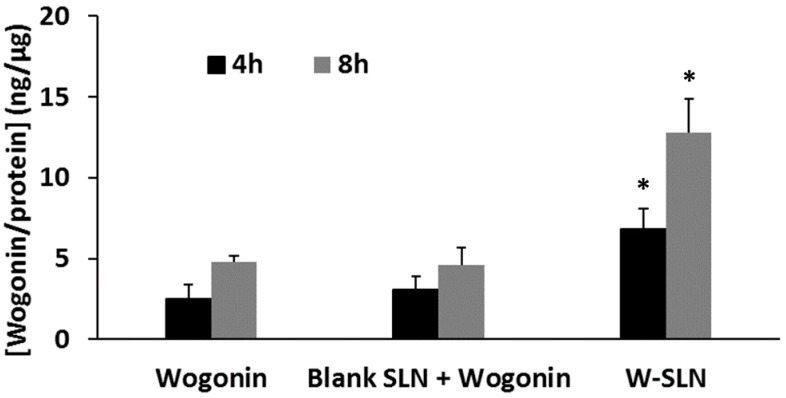
Cellular uptake of wogonin (10 nM) from free wogonin solution, blank SLN with free wogonin, and W-SLN at different time points (4 and 8 h) in MCF-7 cells (*n* = 3, mean ± SD). * *p* > 0.05 shows a significant difference from wogonin.

Figure 6 shows the quantitative cellular uptake of wogonin from different formulations. All of the formulations exhibited a time-dependent cellular uptake of wogonin by MCF-7 cells. The co-treatment of wogonin and blank solid lipid nanoparticle did not enhance cellular uptake of wogonin by MCF-7 cells compared to the wogonin solution. As expected, W-SLN showed the highest cellular uptake of wogonin among the tested formulations regardless of incubation time. At 8 h, W-SLN showed significantly higher uptake of wogonin (12.8 ± 2.1 ng/µg) while wogonin solution and blank SLN with free wogonin exhibited 4.8 ± 0.4 ng/µg and 4.6 ± 1.1 ng/µg, respectively.

**Figure 7:**
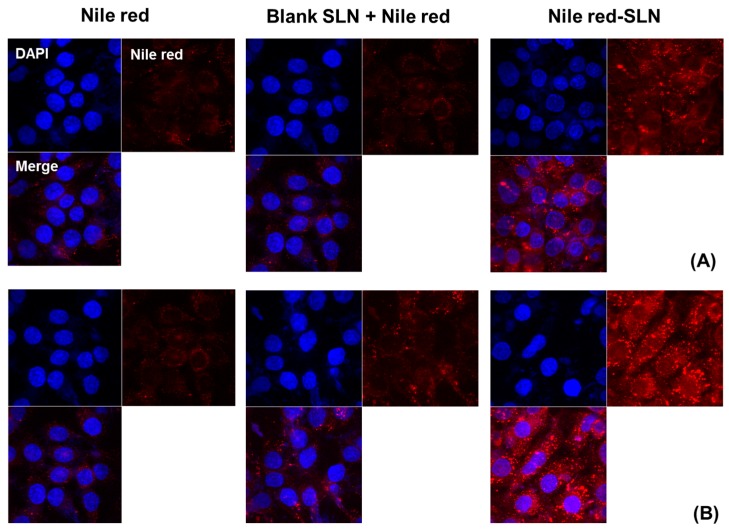
Cellular uptake of Nile red solution, Nile red with blank SLN and Nile red-loaded SLN for (**A**) 4 and (**B**) 8 h of incubation in MCF-7 cells (*n* = 3, mean ± SD). DAPI (4′,6-diamidino-2-phenylindole), Nile red, and Merge represent the nuclear staining (blue), the distribution of Nile red (red), and the merged image, respectively.

For qualitative cellular uptake of SLN, Nile red was encapsulated—instead of wogonin—in SLN. The fluorescence images were taken by confocal microscopy. Similar to the quantitative cellular uptake studies, Nile red-loaded SLN exhibited higher fluorescence than the other formulations in MCF-7 cells. In addition, there was no obvious difference in fluorescence between free Nile red and blank SLN with free Nile red (Figure 7).

### 2.7. Western Blot

**Figure 8:**
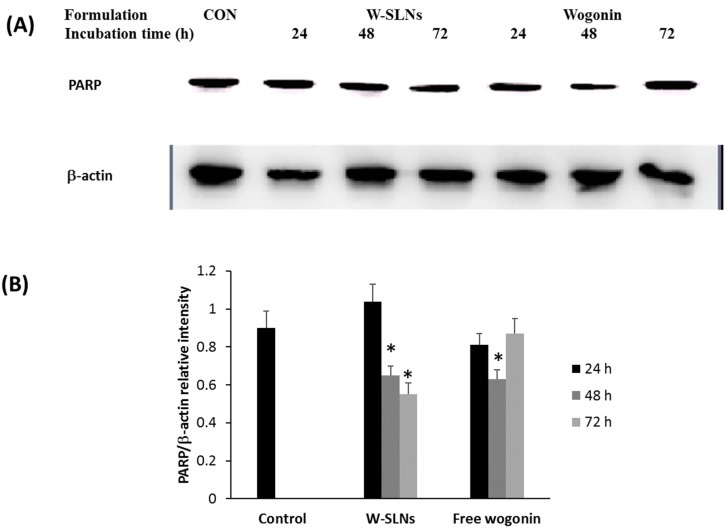
The effect of W-SLN and wogonin on the expressions of poly (ADP-ribose) polymerase (PARP) in MCF-7 cells. (**A**) Western blot assay of PARP and β-actin of MCF-7 cells were incubated with DMEM (CON), W-SLN, and wogonin for 24, 48, and 72 h; (**B**) PARP/β-actin relative intensity of control, W-SLNs, and free wogonin. * *p* > 0.05 shows significant difference with control.

To determine poly (ADP-ribose) polymerase (PARP) concentrations in MCF-7 cells after exposure to the wogonin solution or W-SLN, a Western blot assay was conducted after cells were treated with free wogonin or W-SLN. The results of the Western blot assay for PARP expression levels are shown in Figure 8. The PARP expression was assessed by normalization of the expression of PARP with the expression of β-actin. As shown in Figure 8B, MCF-7 cells expressed PARP with cell medium (control group) [23]. The normalized PARP expression with free wogonin after 24 h and 72 h of incubation did not show a significant difference with that of control while free wogonin (48 h) exhibited significantly reduced PARP expression compared to control. On the other hand, W-SLNs exhibited significantly decreased PARP expression after 48 h and 72 h of incubation. W-SLNs showed a decreasing tendency of PARP expression over time while the PARP expression in MCF-7 cells treated with free wogonin did not decrease continuously.

## 3. Discussion

In this study, wogonin-loaded solid lipid nanoparticles were prepared using a hot sonication method. Although wogonin is a natural compound with known therapeutic effects on a diverse range of cancer cells, its clinical use is limited by its low bioavailability and fast elimination [24]. SLN has been applied to enhance the water solubility and bioavailability of hydrophobic molecules, including natural compounds [25,26]. Therefore, it was hypothesized that encapsulation of wogonin in SLN would provide controlled and sustained release of wogonin for enhanced therapeutic efficacy on breast cancer cells. Since wogonin has shown anticancer effects on various cancer cells, wogonin-loaded particulate systems should take advantage of the enhanced permeability and retention (EPR) effect. The EPR effect is known to be a major mechanism in tumor accumulation of physiologically-based large molecules and small particles (<less than 250 nm) since many solid tumors have unique structural features such as hyper-vasculature and damaged lymphatic drainage [27,28]. All of the particles showed appropriate particle size to take advantage of the EPR effect. After taking into consideration the optimal characteristics of the tested formulations, F2 was chosen to conduct the next studies. Stability of the colloidal system is one of the key factors to be considered. Stability testing revealed superior stability of F2 up to 60 days based on the insignificant changes in particle size, zeta potential, and E.E (%).

The in vitro release studies were conducted in PBS (pH 6.8). The result shows that wogonin in W-SLN exhibited a bi-phase release pattern. It is clear that the encapsulation of wogonin in SLN allowed for sustained and controlled release. The release mechanism of wogonin from W-SLN can be explained by the distribution of wogonin in the lipid matrix. In other words, some wogonin that localized in the surface is related to an initial release burst and the wogonin encapsulated in the lipid core can be released in a sustained manner by slow diffusion through the SLN [29,30,31]. In addition, the hydrophobic nature of wogonin allows it to interact strongly with the hydrophobic lipid matrix, which results in a sustained release of wogonin [32,33,34].

Furthermore, W-SLN showed, by cell inhibition studies, sustained cytotoxicity against cells. The lipid matrix (stearic acid) of the nanoparticles showed no toxicity on MCF-7 cells [35,36]. W-SLN showed continuous cytotoxicity on MCF-7 cells over time while the recovery of cell viability was observed for cells treated with free wogonin. This result indicates that W-SLN showed a sustained proliferation inhibition in MCF-7 cells. The sustained release of wogonin from SLN may enhance the therapeutic efficacy of drugs in vitro and in vivo [37,38,39].

The cellular uptake of different formulations was evaluated in MCF-7 cells. The free wogonin solution exhibited poor cellular uptake of wogonin, while W-SLN was easily taken up by the cells, strongly indicating the SLN resulted in a more extensive and prolonged exposure of wogonin to the cells relative to the wogonin solution. In order to confirm the successful cellular uptake of SLN formulations, Nile red was introduced into SLN instead of wogonin. Since wogonin does not possess autofluorescence, Nile red was chosen as a dye because it is also a hydrophobic molecule with a similar molecular weight. It is highlighted that W-SLN exhibited the highest fluorescence over time, while Nile red solution and blank SLN with Nile red did not exhibit obvious fluorescence over time. This result correlated well with the enhanced cytotoxicity and cellular uptake of wogonin from W-SLN. 

The cell cycle is a major event that leads to cell cleavage and replication. In addition, regulating the cell cycle is an important step in the survival of single cells. The cell cycle is divided into four sequential phases: G1, S, G2, and M. Cancer cells are mainly characterized by the disruption of cell cycle regulation, resulting in dysregulation of cell proliferation. The regulation of the cell cycle is a crucial process in cell survival. For example, regulating the G1/S and G2/M checkpoint enables control of the progression of the cell cycle and, consequently, it can be a strategy for the elimination of cancer cells. Wogonin induces cycle arrest at the G0/G1 phase in diverse types of cancer cells [40,41]. As seen in Figure 5, the cells treated with the wogonin solution or W-SLN exhibited a similar pattern in the G0/G1 phase up to 48 h. MCF-7 cells treated with wogonin had a higher sub G1 phase (apoptosis) than cells treated with W-SLN for 48 h. However, a reverse tendency was shown at 72 h, which correlates with the results of the cell proliferation test. The results obtained indicate a sustained release of wogonin from W-SLN, allowing for the continuous effect of wogonin on the MCF-7 cells. 

Apoptosis is the programmed cell death regulated by different enzymes and genes. PARP, a DNA repair enzyme activated by DNA damage, has been used as a biochemical marker of apoptosis [42]. Therefore, the inhibition of PARP can inhibit tumor growth. In this work, the inhibition level of free wogonin and W-SLN was evaluated for 72 h by Western blot assay. PARP expression with W-SLN was significantly downregulated with increasing time while free wogonin was not able to inhibit PARP over time. This phenomenon is likely due to sustained release of wogonin from W-SLN. Although exposure to a burst amount of wogonin showed immediate therapeutic effect on MCF-7 cells, the effect on MCF-7 cells was not sustained over time. On the other hand, sustained release of wogonin from W-SLN allowed for continuous inhibition of PARP expression in MCF-7 cells over time. Overall, the study demonstrates that W-SLN not only enhances its therapeutic efficiency via inhibiting the cell cycle at the G0/G1 phase, which induces apoptosis, but it also sustains the cytotoxic effect on MCF-7 cells.

## 4. Materials and Methods 

### 4.1. Materials 

Wogonin hydrate, Dynasan 118, and mannitol were obtained from Sigma-Aldrich (Steinheim, Switzerland). Stearic acid and GMS were purchased from Samchun Chemical Co., Ltd. (Pyungtaek, Korea). Lecithin and poloxamer 188 were obtained from Junsei (Tokyo, Japan) and BASF (Ludwigshafen, Germany), respectively. Dialysis membrane (Membra-Cel) was purchased from Viskase, Inc. (Chicago, IL, USA). 

### 4.2. Solubility of Wogonin in Different Solid Lipids

To choose an ideal solid matrix, the solubility of wogonin was measured by melting in stearic acid, GMS, and Dynasan 118. In brief, each lipid (2 g) was melted 10 °C above the melting point of the lipid in a water bath and wogonin (10 mg) was added to the melted lipid until saturation was achieved [42]. The samples were then centrifuged at 15,000 rpm for 10 min and the supernatant (0.5 mL) was suitably diluted with methanol and analyzed by HPLC (Figure S2). 

### 4.3. Preparation of Wogonin-Loaded SLN

Preparation of wogonin-loaded SLN was performed by a modified hot sonication technique (Table 1). Firstly, stearic acid (100 mg) was melted at above melting point (~80 °C). Wogonin (5, 10, or 20 mg) was solubilized in ethanol (0.5 mL) and added dropwise into molten lipid. A mixture of surfactant (75 mg lecithin and 75 mg poloxamer 188) was dispersed in hot deionized water (10 mL, ~80 °C) under sonication for 10 min. Then, the emulsion was quickly cooled in an ice bath to form SLN. Then, mannitol (25 mg) was added to the final solution as a cryoprotectant before freezing. The formulations prepared were lyophilized using a freeze-dryer (FD-1000, EYELA, Tokyo, Japan) for 48 h and stored at 4 °C for further studies.

### 4.4. Encapsulation Efficiency (E.E) (%) 

Ethanol (1 mL) was added to the SLN solution (0.5 mL) in a hot water bath (80 °C) to dissolve the lipid matrix of SLN. After 10 min, the mixture was kept in the fridge (4 °C) to precipitate the lipid matrix. The clear supernatant was filtered using a syringe filter (0.22 µm) to remove precipitated lipid and drug. The filtered solution (20 μL) was analyzed using HPLC system (Agilent 1100, Santa Clara, CA, USA) with a mobile phase (75% methanol and 25% ammonium acetate buffer (pH 4.0) distilled water) at wavelength 270 nm with a 1 mL/min flow rate. The column used was a Hedera ODS-2 column (4.6 mm in diameter and 250 mm length, 5 μm). Limit of detection (LOD) and limit of quantitation (LOQ) were 20 ng/mL and 50 ng/mL, respectively. Coefficient of determination (R2) was 0.9991 within the concentration range of 0.05–500 μg/mL. E.E was calculated as follows:

E.E (%) = measured amount of drug in SLN/weight of the used drug × 100.

### 4.5. Determination of Particle Size and Zeta Potential

The freeze-dried SLN (3 mg) was added to distilled water (3 mL) under sonication for 10 s. Then, dynamic laser scattering analyzer (ELS-8000, Otasuka Electronics, Osaka, Japan) was used to measure particle size. Zeta potential of SLN was measured by a Zetasizer Nano Z (Malven, UK). 

### 4.6. Drug Release Study

The in vitro release profile of wogonin from wogonin solution and W-SLN was determined using a dialysis membrane (MWCO 7k). Wogonin solution was prepared by dissolving wogonin in ethanol. Wogonin solution or W-SLN (20 µg of wogonin) was immersed in PBS (30 mL, pH 7.4, 2.0 wt % BSA). At pre-determined time points, 1 mL of release medium was replaced with fresh medium (1 mL). Wogonin concentration was analyzed by HPLC as mentioned in the Section 4.4.

### 4.7. Cell Studies

#### 4.7.1. Cell Culture

MCF-7 cells were obtained from the American Type Culture Collection (ATCC, Manassas, VA, USA). The cells were cultured in Dulbecco’s modified Eagle’s medium (DMEM) supplemented with 10% fetal bovine serum (FBS) and 100 units/mL penicillin in a humidified atmosphere of 5% CO_2_ at 37 °C.

#### 4.7.2. Cell Growth Inhibition

The cytotoxicity of the wogonin solution and W-SLN against MCF-7 cells was measured using the MTT (3-[4,5-dimethylthiazole-2-yl]-2,5-diphenyltetrazolium bromide) assay. Briefly, 200 µL of MCF-7 cells (5 × 10^4^ cells/mL) was seeded into 96-well plates and incubated for 24 h. Wogonin solution or W-SLN was added and incubated for 24, 48, and 72 h. After that, the medium was discarded and the MTT solution (5 mg/mL, 200 µL/well) was added. After 4 h, the medium was replaced with DMSO (200 µL). A microplate reader (Sunrise, Männedorf, Switzerland) was used to measure the absorbance at 570 nm.

#### 4.7.3. Cellular Uptake

For the quantitative uptake study, MCF-7 cells were seeded in a 6-well plate with a density of 1 × 10^6^ cells per well. After incubation (24 h), the cells were treated with different formulations for 4 or 8 h. After that, the medium containing the formulation was discarded and the cells were washed with cold PBS three times. The cells, then, were lysed using 1% Triton X-100 (400 μL) and the cell lysate was used to determine the total cell protein amount by the BCA assay. The concentration of wogonin in the cell lysate was measured by HPLC as described above.

For the qualitative uptake study, Nile red was encapsulated in SLN. The cells were incubated on a glass-based dish (Thermo Fisher Scientific, Waltham, MA, USA) and observed by confocal laser scanning microscopy (CLSM, Model: LSM5LIVE; Carl Zeiss, Wetzlar, Germany). In the glass-based dish, the MCF-7 cells were seeded at a density of 1 × 10^6^ cells per well in growth medium (1 mL) and incubated for 24 h to allow them to attach to the dish. Then, the cells were treated with the different formulations in growth medium. At predetermined time points (4 or 8 h), the cells were washed three times with cold PBS. Then, cells were stained with 1 μg/mL DAPI in PBS for 3 min and washed twice with PBS. The cells were observed directly under the CLSM.

#### 4.7.4. Cell Cycle Analysis 

After treatment for 24, 48, and 72 h, the cells were collected by centrifuging at 1500 rpm and washed with PBS. Then, PI and ribonuclease were added to stain the cells. Flow cytometer (FACS Calibur flow cytometer; BD Biosciences, San Jose, CA, USA) was used to measure the cell cycle.

#### 4.7.5. Western Blot 

After exposure to different formulations for 24, 48, or 72 h, the formulation was discarded and MCF-7 cells were washed thrice with PBS. The cells were lysed in a mammalian protein extraction reagent (MPER). The cell lysates were centrifuged for 15 min at 15,000 *g* at 4 °C. The soluble fractions were processed for SDS-PAGE at 100 V for 120 min and immunoblotting onto a PVDF membrane was performed using a PowerPacTM HC (BIO-RAD, Hercules, CA, USA). PARP (116 kDa) or β-actin (42 kDa) was detected using PARP antibody (1:1000 dilution, Santa Cruz Biotechnology, Santa Cruz, CA, USA) or β-actin antibody (1:1000 dilution, Cell Signaling Technology, Danvers, MI, USA), followed by a secondary antibody IgG-HRP: sc-2004 (1:4000 dilution).

### 4.8. Statistical Analysis

The Student’s *t*-test was used to compare two different groups of samples. A *p*-value < 0.05 was considered significant. 

## 5. Conclusions

Wogonin was successfully encapsulated in solid lipid nanoparticles using stearic acid as the lipid matrix. The release rate of wogonin from SLN was slower than that of pure wogonin. Furthermore, the W-SLN may be a promising and potent carrier for the delivery of wogonin against the breast cancer cells. Future work will involve the synergistic effects of conventional anticancer drugs and wogonin in the SLN system as wogonin has a unique cytotoxic mechanism.

## Figures and Tables

**Table 1:** Composition of the different wogonin-loaded solid lipid nanoparticles (SLNs).

(Unit: mg)	Formular		
	F1	F2	F3
Wogonin	5	10	20
Stearic acid	100	100	100
Lecithin	75	75	75
Poloxamer 188	75	75	75
Mannitol	25	25	25
Total	280	285	295

**Table 2:** The particle size, polydispersity, and encapsulation efficiency (E.E) of different SLNs. Data are expressed as the mean ± SD (*n* = 3).

Formular	Physicochemical Properties			
	Particle Size (nm)	Polydispersity	E.E. (%)	Zeta Potential (mV)
**F1**	211.6 ± 9.2	0.185 ± 0.012	92.5 ± 3.2	−35.1 ± 5.7
**F2**	225.0 ± 8.3	0.180 ± 0.010	93.2 ± 4.7	−41.7 ± 3.2
**F3**	243.5 ± 8.7	0.254 ± 0.024	78.2 ± 3.8	−43.1 ± 4.1

**Table 3:** Cell cycle distribution of MCF-7 cells treated with wogonin or W-SLN for 24, 48, and 72 h.

Group	Cell Cycle Phase		
(Unit: %)	G_0_/G_1_	S	G_2_/M
Control	68.0	11.0	19.4
wogonin (24 h)	69.5	8.9	19.2
wogonin (48 h)	74.4	6.3	16.1
wogonin (72 h)	72.9	5.4	18.4
W-SLN (24 h)	70.4	7.6	19.9
W-SLN (48 h)	71.4	7.1	21.8
W-SLN (72 h)	83.8	5.5	10.2

## Supplementary Materials

The following are available online at http://www.mdpi.com/2079-4991/8/3/159/s1, Figure S1. Stability test of W-SLN (F2). Particle size, zeta potential and encapsulation efficiency of W-SLN was monitored up to 60 days at room temperature (*n* = 3, mean ± SD). Figure S2. HPLC profile of wogonin for (A) E.E (%) measurement and (B) cellular uptake study.
