# Supplementary material for: Sustained Cytotoxicity of Wogonin on Breast Cancer Cells by Encapsulation in Solid Lipid Nanoparticles
Source: Nanomaterials (Basel). 2018 Mar 13;8(3):159. doi: 10.3390/nano8030159 (PMC5869650; doi:10.3390/nano8030159)
Supplement: Supplementary file 1 [file nanomaterials-08-00159-s001.docx]

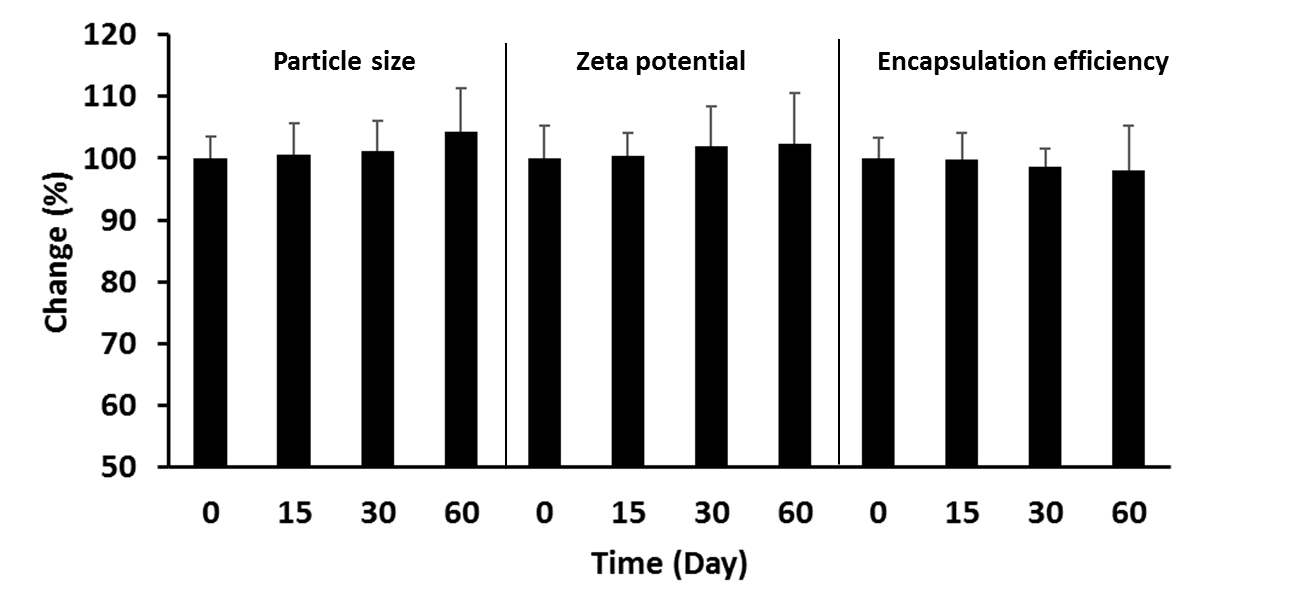


**Figure S1.** Stability test of W-SLN (F2). Particle size, zeta potential and encapsulation efficiency of W-SLN was monitored up to 60 days at room temperature (n = 3, mean ± SD).


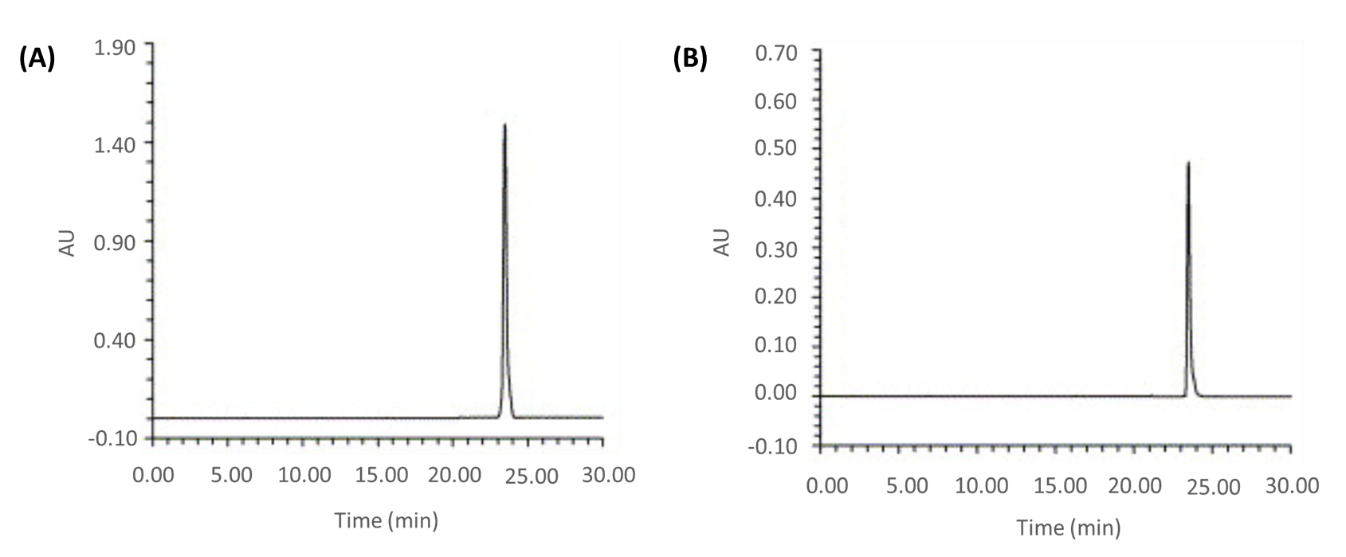


**Figure S2.** HPLC profile of wogonin for (**A**) E.E (%) measurement and (**B**) cellular uptake study.
